# An Estimation of the Numbers of Patients Suitable for Lecanemab Treatment for Alzheimer's Pathology Within Mid and South Essex

**DOI:** 10.1192/bjo.2024.229

**Published:** 2024-08-01

**Authors:** Ivan Shanley, Piyush Pranay, Chitra Dilip, Christopher St Hill, Feena Sebastian

**Affiliations:** Essex Partnership University NHS Foundation Trust, Essex, United Kingdom

## Abstract

**Aims:**

This paper sought to estimate the number of potential candidates per year, within the boundaries of the Mid and South Essex Integrated Care System, for the receipt of Lecanemab, a novel treatment of Alzheimer's pathology.

**Methods:**

One of the four memory assessment services within the region was selected at random, following which all referrals to that service in January and February 2023 were retrieved from the electronic patient record system (n = 45). These records were then screened to assess whether the patient met the criteria for treatment with Lecanemab. The inclusion and exclusion criteria from the original CLARITY-AD phase 3 clinical trial (van Dyck et al., 2022) were combined with those of the Appropriate Use Recommendations released by the Alzheimer's Disease and Related Disorders Therapeutics Work Group (Cummings et al., 2023)^1,2^. Patients could not be identified as certainly suitable for treatment, but simply as potential candidates, as current practice does not include all the necessary investigations to receive the new drug, for example undergoing amyloid PET or CSF testing.

**Results:**

11 of 45 referrals were potential candidates for novel therapeutics (24.4%). Of the 11, 3 were diagnosed with Alzheimer's disease (27%), and 8 with Mild Cognitive Impairment (73%). 8 were male, 3 female, with a mean age of 78 years (range 70 to 87). The mean score on the Addenbrooke's Cognitive Examination III was 82/100. Two patients had co-morbid mental illness, both mixed anxiety and depression, currently in remission. Extrapolating from this rate of eligibility for treatment, it is suggested that approximately 260 patients per year would be eligible for Lecanemab treatment within Mid and South Essex.

**Conclusion:**

This paper estimates that approximately 260 patients per year would be eligible for Lecanemab treatment within Mid and South Essex based on the inclusion and exclusion criteria stated above. This estimate is given with caution, particularly as neither amyloid PET nor CSF testing was performed, and it is still not clear what other stipulations may be made by the UK regulatory bodies (for example the degree of vascular pathology permitted on neuroimaging). This paper does however provide a useful, early estimate of eligibility in order to facilitate planning for potential treatment pathways.

